# Obstacle Avoidance Path Planning for Worm-like Robot Using Bézier Curve

**DOI:** 10.3390/biomimetics6040057

**Published:** 2021-09-30

**Authors:** Yifan Wang, Zehao Liu, Akhil Kandhari, Kathryn A. Daltorio

**Affiliations:** Department of Mechanical and Aerospace Engineering, Case Western Reserve University, Cleveland, OH 44106, USA; yxw780@case.edu (Y.W.); zzl@case.edu (Z.L.); axk751@case.edu (A.K.)

**Keywords:** worm-like peristaltic robots, nonholonomic robots, Bézier curve, obstacle avoidance, path planning simulations, biomimetic locomotion

## Abstract

Worm-like robots have demonstrated great potential in navigating through environments requiring body shape deformation. Some examples include navigating within a network of pipes, crawling through rubble for search and rescue operations, and medical applications such as endoscopy and colonoscopy. In this work, we developed path planning optimization techniques and obstacle avoidance algorithms for the peristaltic method of locomotion of worm-like robots. Based on our previous path generation study using a modified rapidly exploring random tree (RRT), we have further introduced the Bézier curve to allow more path optimization flexibility. Using Bézier curves, the path planner can explore more areas and gain more flexibility to make the path smoother. We have calculated the obstacle avoidance limitations during turning tests for a six-segment robot with the developed path planning algorithm. Based on the results of our robot simulation, we determined a safe turning clearance distance with a six-body diameter between the robot and the obstacles. When the clearance is less than this value, additional methods such as backward locomotion may need to be applied for paths with high obstacle offset. Furthermore, for a worm-like robot, the paths of subsequent segments will be slightly different than the path of the head segment. Here, we show that as the number of segments increases, the differences between the head path and tail path increase, necessitating greater lateral clearance margins.

## 1. Introduction

Worm-like, peristaltic motion enables a robot to use its entire body surface for traction in confined spaces. This type of locomotion enables navigation through environments that other autonomous robots would have difficulty traversing [1,2]. However, the trade-off for worm-like robots is speed. Peristaltic motion waves are composed of small, coordinated body actuations, each of which has limited strain. Thus, a segment’s motion during a single wave (the stroke length) is often small relative to body length. While turning, the stroke length can be further reduced [3], which decreases speed. Thus, understanding and optimizing curved paths is essential for being able to utilize peristaltic motion efficiently in robotics.

For rigid-bodied robots, collision-free trajectory methods have proved reliable both in simulation and in physical systems [4,5]. Specifically, rapidly exploring random tree (RRT) algorithms are heavily used to generate paths for rigid robots [6,7,8]. These algorithms explore random points within a given range and assign a point with minimal cost as the new waypoint towards the goal. For practical applications, path smoothing methods are also critical when the robot is non-holonomic. In other words, a curved path method to connect waypoints needs to be established to prevent sudden directional turning along a path when the robot’s angular acceleration and linear acceleration are limited. In such cases, the Bézier curve is frequently chosen to form smooth paths [9,10,11]. The Bézier curve, which is mathematically defined as Bernstein polynomials, can smoothly connect two endpoints with specific direction requirements, and the curve’s shape can be easily adjusted by modifying its control points [12].

Variants of the planning methods used for rigid robot path generation are being introduced for compliant and multi-segment robot locomotion. For example, open-loop obstacle-aided navigation has been implemented in a soft-growing robot [13]. In this study, the obstacles passively steer the robot, consequently reducing the uncertainty of the robot′s location and directing the robot to targets that do not lie in a straight path from the starting point. Dutta et al. [14] investigated a critical-point bug-based path planning algorithm for a wheeled snake robot. Their robot is rigid but has many degrees of freedom and non-holonomic constraints. With their algorithm, a snake-like robot can find a way to arrive at the goal point while avoiding collisions with multiple static obstacles. While many studies on worm-like robots have been conducted, most of them have focused on locomotion patterns and on decreasing slipping during locomotion [15,16,17,18,19]. A recent study also applied the graph search path-planning algorithm to achieve a breadth-first search [20]. To our knowledge, RRT algorithms have not been implemented for compliant robots to avoid obstacles in path planning.

Planning for worm-like robots presents unique challenges compared to rigid robots [21]. Reachable space is limited. The no-slip constraints at each segment are non-holonomic and can depend on neighboring segments. Furthermore, the structure itself is soft, which can deform the body in difficult-to-predict ways. In addition, unlike a train of segments that follow the same tracks, each segment’s path is slightly different, which can increase complexity because of the number of degrees of freedom. While wheeled robots can be characterized by a minimum turning radius, for worms, the minimum turning radius depends on the wave pattern, current body shape, and length of turn, and other factors [22,23,24,25].

In our previous work, we realized an RRT algorithm-based path planning algorithm with a given start and goal in an obstacle-free environment [6]. This algorithm was applied to the simulation of our group’s worm-like robot structure named Compliant Modular Mesh Worm with steering (CMMWorm-S) [26]. With this path planner, it is possible to calculate the number of waves needed to obtain arbitrary combinations of positions and orientations in space. We further introduced a modified version of the RRT algorithm, the RRT-ellipse method, which made path generation more efficient by reducing the number of iterations needed to find the most feasible path. By the definition formula of an ellipse $\{\begin{array}{c} x=h+a\cdot \cos(t)\\ y=k+b\cdot \sin(t) \end{array},t\in \left(0,2\pi \right]$, for each consecutive two waypoints and their corresponding facing direction, there will be one specific ellipse match. By reversely solving *h*, *k*, *a*, *b*, we obtain the ellipse curve. The set of generated curves therefore smoothly form a path through the given waypoints.

However, the elliptical curve lacks the ability to adjust its shape for the given endpoints. This further reduces the flexibility needed to obtain a more efficient trackable path in an environment with obstacles.

In this article, we develop an efficient and robust obstacle avoidance path planning method for the CMMWorm-S robot as well as for other robots that use peristalsis to locomote around obstacles. We show that switching from elliptical to Bézier curve path generation results in the generated being path more suitable for the robot to follow with less errors. We evaluate this approach by finding paths between two obstacles that create a narrow passageway. If the robot’s original path is aligned with the passageway, then planning is trivial since no adjustments are needed. Specifically, we evaluated passageways that are parallel to the original path but are offset by some distance, akin to offset pipes or channels. We found that as the robot approaches the new passageway (i.e., obstacle clearance decreases), the reachable space is increasingly limited. As a result, the amount of offset is a function of the clearance distance after a critical obstacle clearance, which, for our robot, is about six body diameters. We also compared different robots with varying numbers of segments and found that an increasing number of segments inversely affects turning capabilities. Finally, we conducted an empirical comparison with the physical robot and verified the c offset clearance characteristic we obtained from the simulation.

## 2. Materials and Methods

### 2.1. Worm-Like Robot Structure and Kinematics

**Figure 1:**
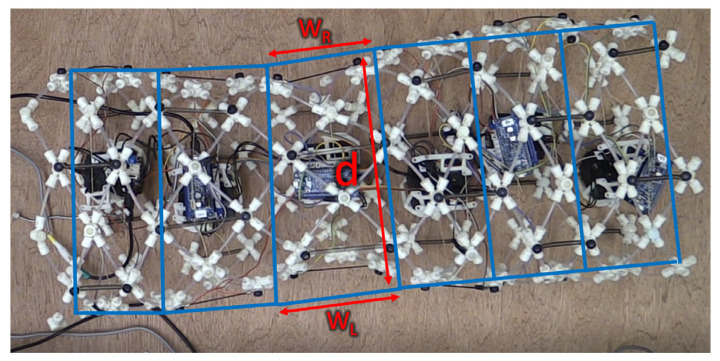
A typical worm-like robot can be simplified as a series of 2D trapezoids (blue lines) applied to Compliant Modular Mesh Worm with Steering robot (CMMWorm-S) as viewed from the top [18].

This research aims to generate efficient path planning for worm-like robots to navigate around obstacles with a designated peristaltic turning gait. In particular, our research is based on the simulation of our worm-like robot: Compliant Modular Mesh Worm-like Robot with Steering (CMMWorm-S robot) [18], as shown in Figure 1. The CMMWorm-S mimics the locomotion pattern of an earthworm using its deformable mesh structure.

We have previously developed a kinematic model [3] for this robot in which we assumed that each segment (the region closest to a set of cable actuators) is a trapezoid. Two sides of the trapezoid have equal length d, which is equal to the diameter. The left side of the trapezoid is determined by the left cable and has length W_L,_ and the right side is controlled by the right cable and has length R_L._ This model proved useful for our peristaltic robot when considering turns [25], but this should also apply to other worm-like robots. Thus, by adjusting the robot-related parameters, the methods introduced in this paper and their corresponding results can be applied to different types of worm-like robots that locomote using peristalsis.

The robot body begins in a cylindrical shape with a diameter of 20 cm and can be viewed as six independent segments whose shape (diameter and left/right width) can be controlled by their corresponding actuator pairs. With preprogrammed code, the actuators will control these segments to expand or contract in sequence to locomote by forming a peristaltic wave [24]. In this paper, we primarily use a 2 × 1 wave pattern for locomotion. Such pattern indicates two continuous segments deforming at the same time (front segment contracts while rear segment expands), and only one pair of this deformation is actuated at any given time. In the simulation, each segment is represented by trapezoids (blue lines in Figure 1) with the parameters *d*, W_R,_ and W_L_. The diameter (*d*) is simplified as a preset constant value. The left/right width (and W_L_) of these trapezoids defines the robot’s present shape, and their changes during time represent the locomotion of the worm-like robot.

**Figure 2:**
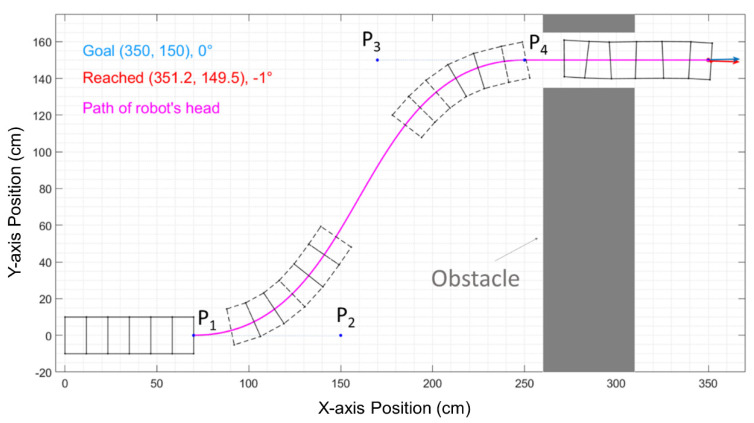
A worm-like robot passes through the obstacles solved by the Bézier curve path algorithm in our MATLAB simulation. The shape of the curve is controlled by points $P_{2}$ and $P_{3}$.

Our simulation environment is set up with our robot placed at the left side of obstacles and facing horizontally from left to right. All of the results in this paper are independent of the choice of coordinate reference frame, but for the convenience of comparison, the robot′s initial configuration is always set so that its rear edge is on the y-axis and its centerline is on the x-axis, as shown in Figure 2. Two rectangular obstacles are also shown in Figure 2. The obstacles form a passage between the robot’s start position and the goal. The passage′s width is 30 cm, which enables 25% diameter clearance on either side.

### 2.2. Bézier Curves

To improve on the path generation algorithms previously published by our group [6], we use cubic order Bézier curves to generate a smooth path for CMMWorm-S. A cubic order Bézier curve is shown as the purple curve from $P_{1}$ to $P_{4}$ in Figure 2. The parametric equation of the explicit form of a cubic order Bézier curve (B(t)) can be expressed as follows:

(1) $$B\left(t\right)={\left(1-t\right)}^{3}P_{1}+3{\left(1-t\right)}^{2}tP_{2}+3\left(1-t\right)t^{2}P_{3}+t^{3}P_{4},\;t\in \left[0,1\right]$$

where $P_{1}$, $P_{2}$, $P_{3}$, and $P_{4}$ are designated controlling points in Cartesian coordinates. By increasing variable *t* from 0 to 1, the path defined by *B*(*t*) will form the Bézier curve from $P_{1}$ to $P_{4}$, as shown in Figure 2. We name the vectors from $P_{1}$ to $P_{2}$ as the starting control vector $V_{sc}$_,_ and name the vectors from $P_{3}$ to $P_{4}$ as the finishing control vector $V_{fc}$.

For curves to be applied to the smooth path generator, the following requirements must be matched: (1) the given start point and finish point must be the two endpoints of the curve. (2) The curve must be tangential to the corresponding given directions at the start point and finish point. Such directions are either from the path planning configuration (initial/goal robot facing direction) or from the previous curve’s facing direction at its current start point.

The characteristic of the Bézier curve makes it simple to fulfill these requirements. From the equation, we can determine that the curve will always start at $P_{1}$ and end at $P_{4}$. The curve will always be tangential to $V_{sc}$ and $V_{fc}$ at $P_{1}$ and

$$P_{4}$$,

respectively. By setting $P_{1}$ and $P_{4}$’s coordinates as the start/ending point and by setting $V_{sc}$ and $V_{fc}$ to the required directions, the curve can be applied smoothly to connect the two waypoints generated by the path planning algorithm. The derivative of Equation (1) is also continuous, which indicates that there is no sudden turning along the applied curve. Additionally, by changing the length of $V_{sc}$ and $V_{fc}$, we can adjust the curve’s shape for optimization while ensuring the matching of the requirements.

### 2.3. Path Generation with Obstacle Avoidance

Path generation is completed by first finding several critical waypoints and then by generating viable curves and sequentially connecting them. The algorithm will repeat this search until a path is formed by those curves that successfully connects the initial position and goal position without colliding with any obstacles. Optimization is then conducted to reduce the total path length.

**Figure 3:**
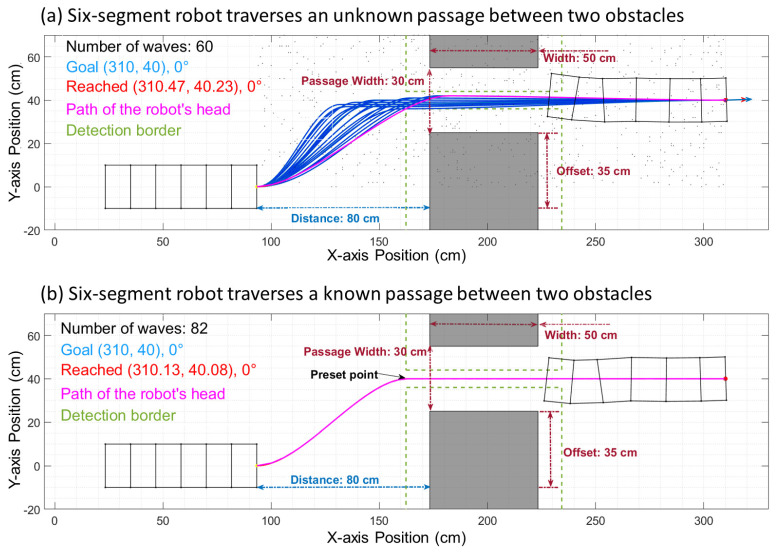
The six-segment robot passes through the obstacles that are arranged to form an offset passage. The dark rectangles represent the obstacles, and the green dashed lines are the detection borders. The gray dots in the background of (**b**) are the random waypoints used to search the whole space. The blue curves are the possible paths to the goal. Additionally, the pink curve is the final path that the robot chose. (**a**) The robot follows the path, successfully traverses an unknown passage between the obstacles, and reaches the goal. (**b**) The robot traverses a known passage with a preset waypoint.

The waypoints can either be (1) generated randomly or (2) be heuristically determined. In the first case, when no a priori information is given to the algorithm and the waypoints needs to be discovered, the algorithm will pick random points from the whole space (as in Figure 3a) and generate the Bézier curve paths with a random control vector length through those points. In this paper, two obstacles are placed to form a passage. Therefore, we use the heuristic that a waypoint at the center entrance of the passage is usually effective, as shown in Figure 3b.

After a potential path has been generated, the algorithm checks collisions. We calculate the collisions by adding a clearance margin to the obstacle borders. Then, we use the MathWorks intersection function for polyshapes to identify if there is an intersection between the path and the shape defined by obstacles. Since the CMMWorm’s radius is 10 cm, we make the collision detection margin 10% larger than the robot′s diameter (11 cm).

In order to optimize the final result, the path generation and collision checking process will repeat with varied parameters and produce different non-colliding paths. The path with the minimal total length is considered the optimal path. In Section 3.1, we will demonstrate that this approach decreases the total waves used for travel even, though due to the complex kinetics of peristalsis, the relationship between the path length and total waves is non-linear.

Based on Equation (1), we can calculate the length of each Bézier curve ***S*** using the following equation:

(2) $$S=\int_{0}^{1}\sqrt{{\dot{x}}^{2}\left(t\right)+{\dot{y}}^{2}\left(t\right)}\;dt,\;t\in \left[0,1\right]$$

where $\dot{x}$ and $\dot{y}$ are the first derivative of the curve’s coordinates with respect to time.

The length equation is an elliptic integral that cannot be expressed in fundamental functions. Therefore, instead of solving for the analytical solution, we use cumulative trapezoidal numerical integration to calculate an approximation of the arc length:

(3) $$S=\int_{0}^{1}B\left(t\right)dt\approx \overset{N}{\underset{i=1}{\sum }}\frac{T}{2N}\left[B\left(\frac{i-1}{N}\right)+B\left(\frac{i}{N}\right)\right],t\in \left[0,1\right]$$

where *N* is an artificially assigned value to represent the total iteration. By increasing *N*, the integration result can be more accurate. *B(t)* is the equation of integrated curve equation.

After calculating the length of the path, we can also compute the how many waves a path has. Our previous work created a kinematic model of the worm-like robot used in this study [18] and an algorithm to allow the robot to follow the path [6]. Based on previous work, we can generate the whole movement of the robot by following the path and can obtain the number of waves. For each wave, the second segment deforms so that the center of the first segment (the head of the robot) follows the path. Then, a 2 × 1 peristaltic wave travels backwards to the remaining segments so that the robot follows no-slip kinematic constraints. Each wave is counted until the robot reaches the goal. The detailed realization is described in Appendix A.

### 2.4. Analysis of Limiting Factors: Radius, Offset, and Clearance

The passage is characterized by the location (offset in y-axis, and clearance in x-axis) relative to the original orientation of the robot. In other words, a passage with a high offset will require more turning to be accommodated, and a passage with greater clearance will have more room to make the required turn.

In previous work, we determined a safe turning radius for stable reorienting turns of 77 cm [3]. Attempting to keep turning at a tighter radius for many waves can result in stall conditions in which the waves make smaller and smaller progress. Thus, all turns were limited to 77 cm radius—the limit of the tightest possible complete circle. However, because the radius of a Bézier curve is always varying here, we show how worm-like robots can take tighter turns over short distances with Bézier curves.

**Figure 4:**
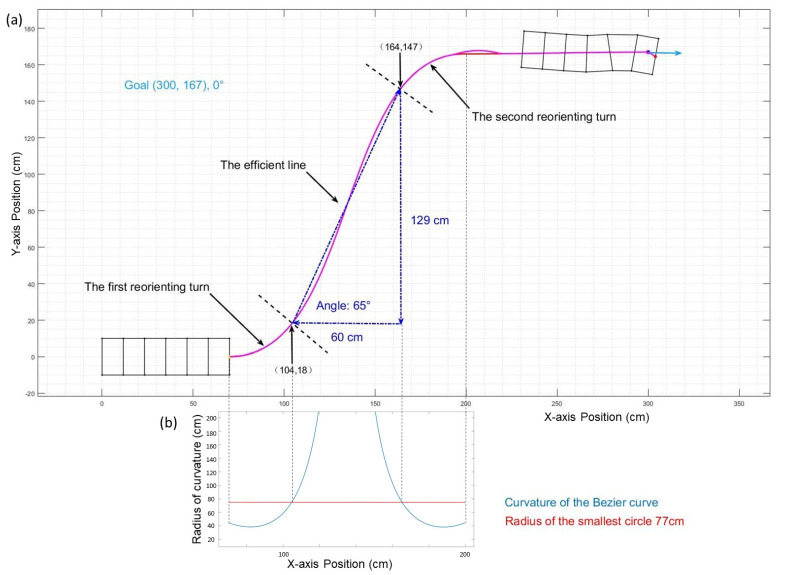
(**a**) The whole path (pink line) is separated into three parts. The first reorienting turn is from the beginning to (104, 18), the efficient line is from (104, 18) to (164, 147) (dashed line), and the second reorienting turn is from (164, 147) to the goal point (300, 167). (**b**) The path curve’s corresponding curvature radius with respect to x position.

To make comparisons with prior turning radii, we visualize the Bézier curve decomposed into three parts: a first reorienting turn, an efficient line, and a second reorienting turn. How the boundaries are determined is based on whether the path’s radius of curvature is larger than the robot’s safe turning radius, as shown in Figure 4. During the first reorienting turn, the robot needs to turn in a direction that is aimed at the obstacles or passage. Then, the efficient line section has a relatively larger curvature radius, allowing the robot to move forward with greater efficiency. In the second reorienting turn, the robot needs to turn to by a certain degree again and reorient itself to pass through the obstacles. The proportion of these three parts affects the robot′s turning clearance. To avoid stall status during the first and second reorienting turn, the direction of the head of the robot when entering the efficient line section is limited. This factor will also influence the maximum obstacle offset that the robot can pass.

## 3. Results

### 3.1. Path Improvements by Bézier Curves Application

**Figure 5:**
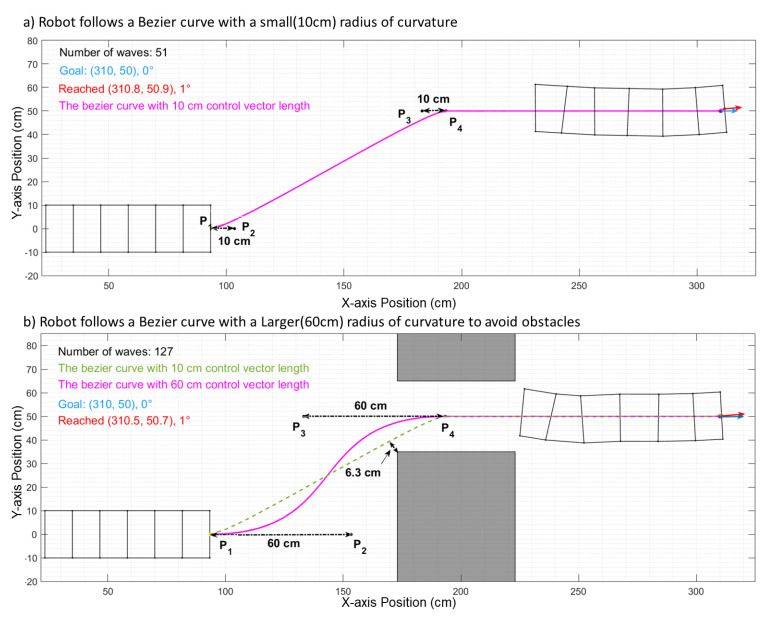
Comparisons of different Bézier curves to reach the same goal in different situations. The Bézier curve of case (**a**) is more linear than that in case (**b**). (**a**) The length of the control vectors is 10 cm. It takes 51 waves for the robot to reach the goal. (**b**) Due to obstacles, the Bézier curve requires a larger turning radius for better passing ability. The length of the control vectors is 60 cm. It takes 127 waves for the robot to reach the goal. For reference, the green dotted line in (**b**) is the path in (**a**).

Bézier curves enable the important ability of adjusting the shape of a curve while matching the starting and finishing configuration and can help optimize the path for different environments with identical starts and goals. By implementing this method, we have conducted related tests in simulation, and the results are compared to those from our previous work using an elliptic path. During the test, we set the start and goal directions horizontally rightward. Therefore, the angles of the control vectors against the x-axis were both 0, as shown in Figure 5. In Figure 5a, the most efficient path to get to the goal configuration (position and orientation) is formed by small control vectors (10 cm and 10 cm). It takes 51 waves (total length of 229 cm) for the robot to reach the goal. In Figure 5b, obstacles are added (gray rectangles). The previous efficient path from (a) (green dashed line) only has 6.3 cm of space available to clear the obstacles, which is smaller than the robot’s radius (10 cm). As such, the optimized path needs to be adjusted (the solid pink line) to avoid the collision. Such an adjustment can be made by increasing the control vectors (60 cm and 60 cm), resulting in a larger turning radius. The optimized path now uses 127 waves (total length of 234 cm) to reach the goal due to the increased environment complexity. The forward max motion per step is reduced when turning due to the kinematics of the robot [3]. Therefore, the total number of waves increases more than the length of the total path does.

**Figure 6:**
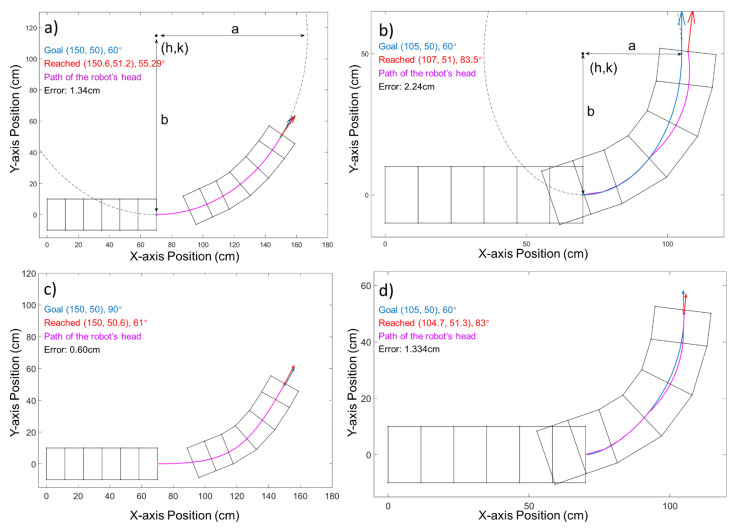
Comparisons between elliptical path generation and Bézier curve path; blue dot and arrow are the desired position and orientation; red dot and arrow are the simulated robot′s reached position. The blue curve is the generated ellipse, and pink is the executed path. (**a**) Blue and red arrows almost coincide, which shows that the robot has successfully reached its goal. (**b**) The robot fails to follow the ellipse; thus, the pink and blue curves do not coincide because the elliptical axis length in the lateral direction is too small. (**c**) The robot follows the Bézier curve and reaches the goal with a much smaller error compared to (**a**). (**d**) The robot deviates from the path but corrects the error to reach the goal.

**Figure 7:**
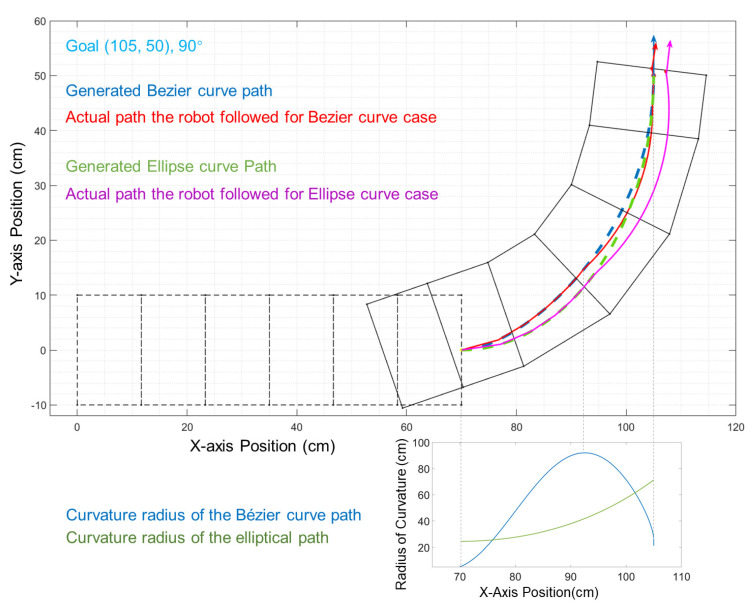
Comparisons between the elliptical path and the Bézier curve paths. The blue curve is the Bézier curve, and the pink is the executed path. The dotted green line is the previous elliptical path. The below figure shows the curvature radius of both paths.

In our previous work, we tested the robot′s performance with different elliptical paths [6]. Using the elliptical curves, for a given set of start and goal configurations, there would be at most one corresponding elliptical path available. Such a relation leaves no space to optimize the path shape between two waypoints. Additionally, given the structural constraints of our soft worm-like robot, there is a limitation on the turning angle, and it can only follow a curve within a given curvature. As shown in Figure 6a,b, when the ellipse path has a curve radius that is too large for the robot to reach, there will be a large error (2.24 cm) at goal. The Bézier curves allow the robot to follow the path accurately by adjusting the control vectors to better fit the robot′s kinematic constraints. In our test, the positional error of the Bézier curve application can be less than half (0.60 cm) of the error of the ellipse path (1.34 cm), as shown in Figure 6a,c. the curvature radius for the Bézier curve can be adjusted to both increase and then decrease rapidly. With such an adjustment, the period where the robot cannot follow the path will be shorter and will enable the robot to further eliminate the accumulated displacement error before reaching its goal, as shown in Figure 6d and Figure 7. With all of these features, the Bézier curve shows an advantage in terms of trackable path generation and error reduction.

### 3.2. Offset Clearance Limitations for Six-Segment Robot

**Figure 8:**
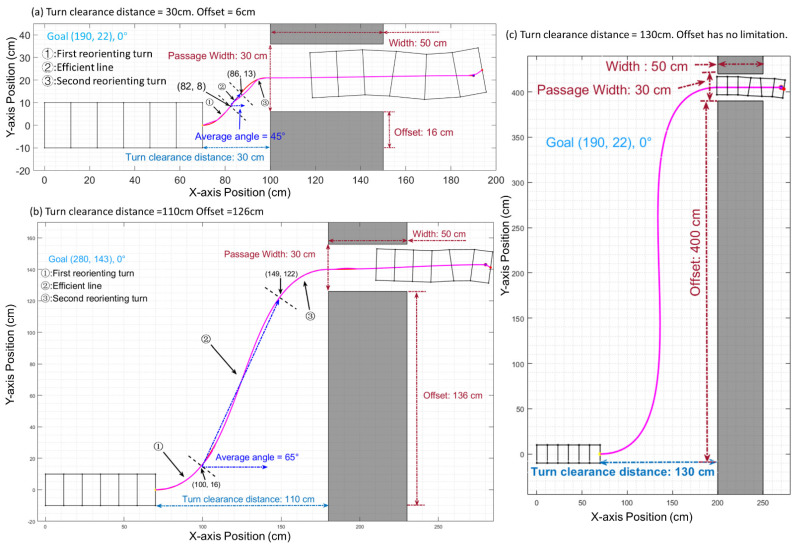
As the robot moves farther from the obstacle, the maximal passable offset dramatically increases. In case (**a**), the distance is 30 cm, and the robot can overcome an obstacle with a 16 cm offset. In case (**b**), the distance becomes 110 cm; the robot can overcome a 136 cm offset obstacle. In case (**c**), the distance is 130 cm; the robot can overcome any obstacle of any offset (400 cm offset shown as example).

Our research establishes a relationship between the maximal passable value of the obstacle offset (distance between obstacle edge and robot’s central axis) and turn clearance distance (distance between obstacle edge and robot’s front edge in the axial direction). Figure 8 shows three cases of such relationship. In Figure 8a, the obstacles are remarkably close (30 cm) to the robot. The length of the efficient line is only 1/4 of the first reorienting turn section. The angle of the efficient line can only reach 45 degrees due to there being insufficient space for the robot to turn at the max angle. In this case, the maximum offset of the obstacle that the robot can pass is 16 cm along the y-axis. In Figure 8b, the obstacles are placed further away (110 cm). The increased distance gives the robot more room to turn at a larger angle, and the efficient line section can take up a large proportion of the whole path in terms of distance. The angle of the efficient line is 67 degrees, and the length along the x-axis for the efficient line section is 1.6 times that of the first reorientation section, and the robot can avoid an obstacle with a 136 cm offset along the y-axis. In Figure 8c, when the distance is larger than 118 cm, the distance is big enough to allow the robot to move vertically, making the robot able to overcome an obstacle of any offset.

**Figure 9:**
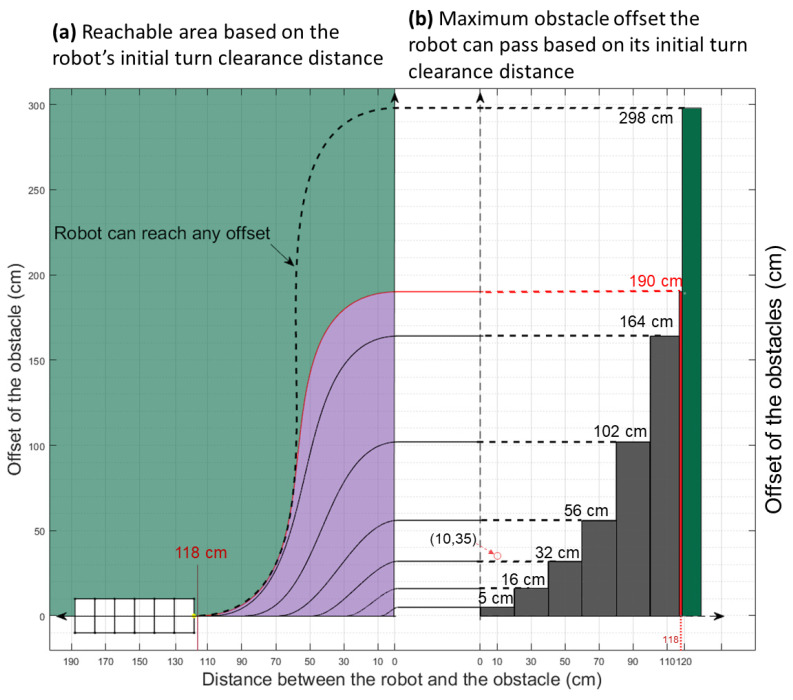
The turn clearance limitations for a six-segment robot shows how much space the robot has to turn with respect to its initial distance to the obstacle (10 cm, 30 cm, 50 cm, etc.). The passing ability increases as the distance between the obstacles and the robot increases. (**a**) shows the boundary paths of the robot and their corresponding reachable area. (**b**) Max offsets of passable obstacles in respect to different initial starting clearances. Note that the obstacle configuration in Figure 10 is shown by the red circle.

Thus, we are able to show the relationship between the limiting offset for a particular clearance. Setting the obstacles at intervals of 10 cm along the x-axis, we find the limiting offset along the y-axis. In this test, we set the waypoint at the center of the entrance of the passage, as shown in Figure 8. The control vector lengths are the same, i.e., the length of $V_{sc}$ is equal to the length of $V_{fc}$. The range of the vector length is limited to between 10% to 50% of the distance between the starting point to the ending point during the random path generation process. As the offset clearance becomes larger, the size of the offset will sharply increase. This emphasizes the importance of planning. Figure 9a shows several potential robot paths and obstacles. The detailed realization is shown in Appendix B.

### 3.3. Backward Movement

**Figure 10:**
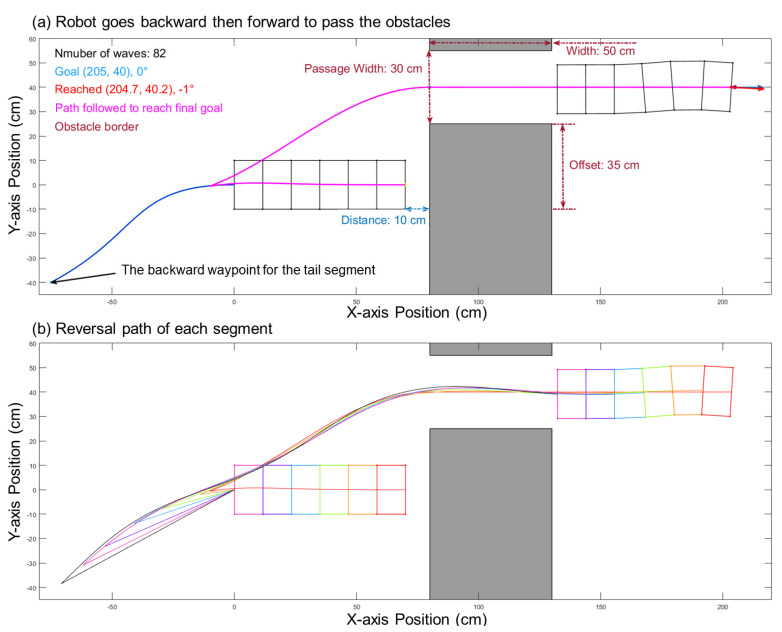
Backward motion is needed if the obstacle is too close. Here, the distance between the obstacles and the robot is 10 cm, and the offset is 35 cm, which is outside the range defined in previous figure (see dot). Therefore, it has to go backward first and then go forward. In (**a**), the robot follows the paths with a backward path, successfully passes through the obstacles, and reaches the goal. (**b**) shows the paths of each segment in different colors.

For the case of Figure 8a, when the obstacle with a large offset is too close to the robot, the robot will not be able to pass the obstacle using forward locomotion. However, enabling backward movement is an available approach to solve this issue. In such a case, the robot moves backward to create enough space and then moves forward with a generated path to pass through the obstacles. To generate the backward Bézier curve, we need to find a goal point on the line passing through the center of the robot’s head and the center of the passage’s entrance. The direction is from the entrance’s center to the robot’s center. Then, we can obtain a backward Bézier curve with the center of the robot’s tail and the backward goal point. After the robot completes the backward motion, the algorithm will continue with forward path planning with the robot’s new position as the starting point, as shown in Figure 10. Using the limitations shown in Figure 9, we can determine whether backward movement is needed and what how far backward the robot needs to move in a particular situation. Specifically, for our robot, if the robot has a distance six-body diameters before the obstacle, this is a safe turning clearance distance for any obstacle offset, and this is the maximum distance required to move backwards.

### 3.4. The Influence on Turning by the Total Number of Segments

**Figure 11:**
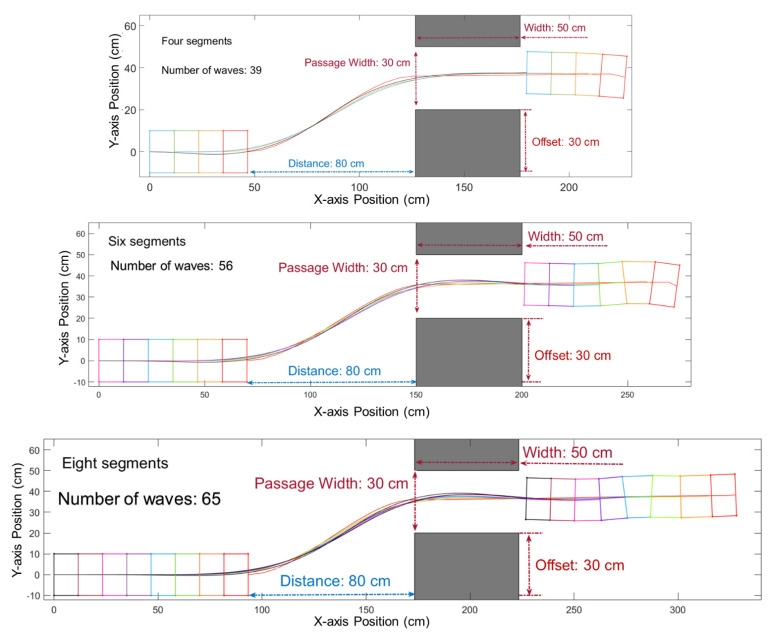
Robots with four, six, and eight segments passing through the same obstacles. The number of waves increases with the increasing number of segments in order for the entire body of the robot to move through the obstacle.

Our research also tested how the increasing number of segments affects the turning path planning, and the simulated result is shown in Figure 11.

Increasing the number of segments increases the path error among segments. According to our simulation of robots with 4, 6, and 8 segments, the path errors among segments increase with the increasing number of segments. The largest error is the error between the first and last segments. Such errors vary while the robot is progressing along its path. We called the largest error along the whole path on the left side L-error and the right side one R-error. Additional clearance margins may need to be given for additional segments.

**Figure 12:**
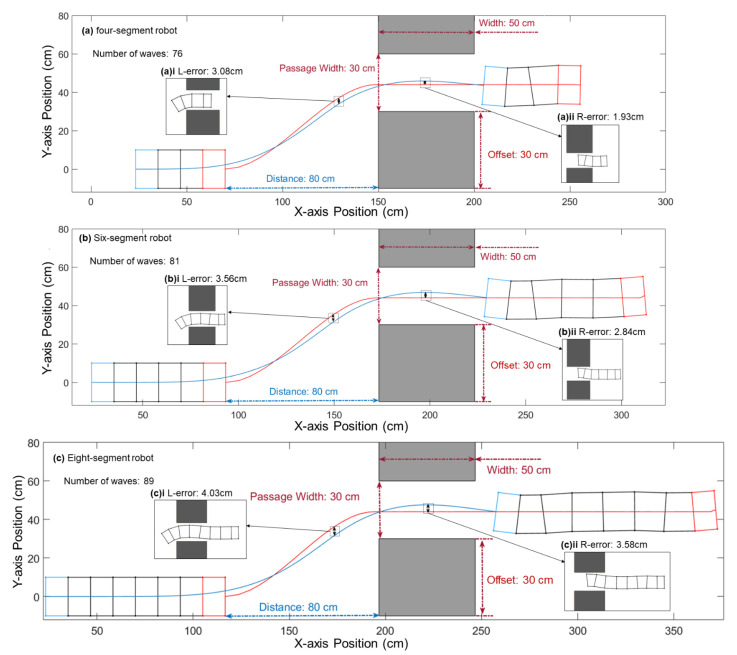
Robots with four, six, and eight segments pass through the same obstacles. The errors increase with the number of segments. The largest L-error and R-error for the four-segment robot (**a**) are 3.08 and 1.93. The largest L-error and R-error for the six-segment robot (**b**) are 3.56 and 2.84, and for the robot with eight segments (**c**), the largest L-error is 4.03, and the largest R-error is 3.58. The two insets show the robots′ positions and gestures when they are at the L-error and R-error. (**a**) i, (**b**) i, and (**c**) i are the waves with the maximum L-error. (**a**) ii, (**b**) ii, and (**c**) ii are the waves with the maximum R-error.

Figure 12 shows the errors between the last segment′s path and the first segment′s path in the 4, 6, and 8 segments cases. The rear part of the robot could collide with the obstacle if the passage becomes narrower or wider. If the passage becomes narrower, the L-error may cause a collision with the left side of the bottom obstacle in the robot′s approach phase, and the R-error may cause a collision with the bottom of the upper obstacle in the passing phase of the robot. Consider an example for the eight-segment robot: if the distance between the robot and the obstacle is 22 cm smaller or if the width of the passage is 1.5 cm smaller, there would be a collision.

The four-segment robot′s L-error is 15.4% of its diameter, it is 17.8% for the six-segment robot, and it is 20.2% for the eight-segment robot. The four-segment robot′s R-error is 9.6% of its diameter, and it is 14.2% for the six-segment robot and 17.9% for the eight-segment robot. The comparison is shown in Table 1.

### 3.5. Empirical Comparison with Physical Robot

Finally, we made an empirical comparison with our physical robot. We tested the robot′s offset clearance limitations in a specific situation where the distance between the robot and the obstacle is 70 cm.

#### 3.5.1. Differences between the Simulation and the Physical Robot

As we mentioned in Section 2.1, the simulation did not consider the slip of the robot. However, in the physical situation, the slip is relatively common in the robot′s movement, and the actual elongations are different from the simulation results. In this case, we needed to calibrate simulation data and make the simulation more practical.

The diameter of the physical robot is 11 cm. The maximum elongation of each segment is 17 cm, and the minimum contraction of each segment is 9.5 cm. The parameters of the physical robot are slightly different from the previous simulation in order to complete the simulation with the physical robot, so we updated the parameters in the algorithm.

**Figure 13:**
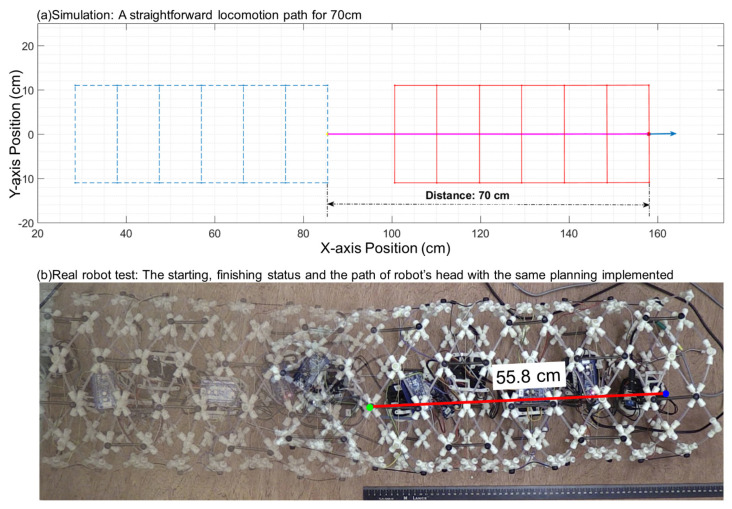
(**a**) a straightforward locomotion path for a 70 cm straightforward generated by the simulation. (**b**) Start (green dot) and finish (blue) position of a real six segment worm-like robot applying the same algorithm. The red line shows the total movement (55.8 cm), which is 80% of the simulation result.

First, we tested a 70 cm straightforward movement with a simulation and a physical worm-like robot to compare the differences between them. The physical robot′s moving distance is 55.8 cm, which is 80% of the simulation result, which is shown in Figure 13.

#### 3.5.2. Offset Clearance Limitations of the Physical Robot

Next, we compared the differences between the physical robot application and the simulation in a turning process and acquired the offset clearance limitation of the physical robot. We tested the offset clearance limitation of the physical robot at a 70 cm distance.

**Figure 14:**
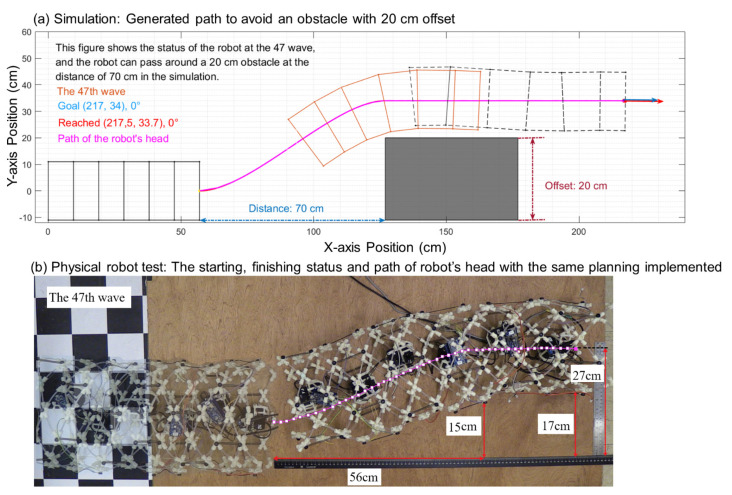
(**a**) simulated robot and obstacle avoidance path. The total displacement is 70 cm on the x-axis and 20 cm on the y-axis (**b**) The starting and finishing status of the physical robot with the same path implemented.

Due to the complexity of the physical environment, structural and dynamical errors, slipping, and spinning is observed during the tested robot’s locomotion. In the simulation, a robot that starts at (57, 0) can reach the goal point at (217, 34) and pass over an obstacle that is 20 cm in width. In the physical robot experiment, the robot finally reached (193.3, 27.3), which is 85.2% of the simulation result in the x-axis direction and 81.1% in the y-axis direction. The result indicated that the physical robot could pass over a 15 cm obstacle at 56 cm, as shown in Figure 14.

The result from the physical robot shows the difference and relationship between the simulation and the physical truth. The path algorithm in our simulation can be implemented with physical robots to perform a similar path pattern to reach the goal and to avoid obstacles. However, corrections need to be applied to the simulation data to compensate for the locomotion errors between simulation and physical movement due to the robot’s configuration measurement error and slipping. The turn clearance distance changed because the distance at which the physical robot travels along the x-axis is 85% of that of the simulation. In the physical experiment, the turn clearance distance changed from 70 cm to 56 cm, and the offset of the obstacle is 15 cm, as shown in Figure 14b. Based on the test result, the final physical locomotion of the test robot is about 80% of our non-calibrated simulation result (the physical experiment also showed that the slipping condition is different between the x-direction and y-direction).

**Figure 15:**
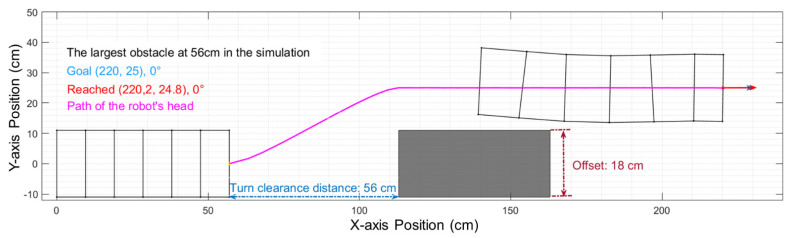
With the application of an 80% scale factor for correction, the simulation can more accurately predict the results of the real-world implementation.

We then recommitted the test with an 80% scale factor for correction. After correction, the offset of the obstacle in the simulation was adjusted to 18 cm, and the turn clearance distance was adjusted to 56 cm, As shown in Figure 15, the offset difference between the simulation and the real-world test has been reduced by half (3 cm).

## 4. Discussion

We have developed an obstacle-avoiding path planning algorithm to ensure that a worm-like robot can pass around obstacles in different situations, with an understanding of the robot′s offset clearance limitation.

There are two major applications for this research. The first one is static path planning for the robot under a known and stable situation. The second application is real-time path planning; the situation is unknown and changeable.

For static path planning, the environmental information is known [27]. The algorithm will need to find a path through the known obstacles. In such a case, we assume that we can obtain information about obstacles in advance or obtain the information from sources outside of the robot, e.g., with an aerial view camera. With this information, the algorithm can generate an appropriate path for the robot. For the static case, the computation time is less important since we can allow the robot to move after all the computation is completed.

For real-time obstacle avoidance path planning, the algorithm can adapt to changes in the environment and can create progress updates. To achieve real-time path planning, the robot should continuously update the information from the surroundings. Options include applying anterior mounted cameras in vine robots [28] and onboard proximity sensors, such as what we previously demonstrated on our robot [17,29]. Laser radar, such as in [30], improves accuracy and multiple cameras, such as in [31], can provide an enhanced shape resolution and refresh rate.

The current computational time cost for a long-distance path generation (50 times of robot total length) may be a challenge for real-time execution. However, choosing a close goal will reduce the computation time. Moreover, for a slowly changing environment, when considering the limited speed of a worm-like robot, even a slow algorithm can have enough time to generate the path for the subsequent few waves.

In the future, comparing different wave patterns may enable better turning. Our work is based on a 2 × 1 wave mode of motion, and this is the simplest method of movement for a physical robot. An actual earthworm or other soft animals can vary the patterns of coordination used for locomotion, and our previous study also indicates performance improvements by optimizing this wave pattern [21]. We can find inspiration from these animals and apply their modes of motion to our worm-like robot. For example, a worm-like robot can use a 3 × 1 and 3 × 2 wave mode to move [18]. In the future, more work will need to be conducted in order to create a more inclusive simulation.

Turn clearance limitations can be important for the practical design of worm-like robots. If six diameters are required to navigate an arbitrarily large offset, this suggests that the robot needs to be able to detect obstacles that are far ahead at the very least. Furthermore, if the robot pathways do not have sufficient open space around them (for example in the case of two pipes that may have shifted relative to each other) a no-slip turn may not be possible between the two paths. Our work shows empirical results for a robot with one set of dimensions but with more segments moving at once using 3 × 1, but more waves can enable tighter turns if the robot stiffness is sufficient. Furthermore, it is possible to take advantage of a slip in some environments.

Since our robot is soft, its low mass and compliant body make its collisions with obstacles inherently safe. Although these collisions will consume more energy, the collisions may sometimes be beneficial. There is some research regarding using obstacles to the benefit of the robot. For example, a snake robot could actively use obstacles to propel itself forward [32]. For worm-like robots, we could also use the benefits of the interactions with the obstacles. In some situations, these interactions could allow us to make an easier turn, such as in a pipe, by using the obstacles′ reaction force. Path planning with the interaction between the robot and obstacles will be an important part of future work.

Understanding the effect of slip and interaction with obstacles will help such robots efficiently align their bodies for the next step of a process and will guide and benefit worm-like robot design to improve their maneuverability.

## 5. Conclusions

Our work aimed to build and apply obstacle avoidance path planning for worm-like robots and provide inspiration and guidance for similar robots using soft body locomotion. In this work, we improved the path-generating algorithm by changing the elliptical curve to the Bézier curve (Figure 3). The Bézier curve gave us the ability to change and optimize the shape of the curve while matching the starting and finishing configuration. This ensured a great advantage of the flexibility of Bézier curves in handling different situations and environmental requirements compared to the previous ellipse path. With the advantages of the Bézier curve, the new path planner can generate smoother and more flexible paths (Figure 5). With the Bézier curve, the robot can reach the goal point with smaller errors compared to the ellipse curve (Figure 6). To find the offset clearance characteristic, we divided the whole path into three parts (Figure 4). The first reorienting turn was directed toward the passage at the beginning of the path; the efficient line has a relatively larger curvature radius in the middle, and the second reorienting turn is oriented to pass through the obstacles. These three parts help us understand the relationship between the horizontal advance distance and vertical advance distance (Figure 8). Based on this algorithm, we were able to determine the worm-like robot′s offset clearance and find the limiting size of the obstacles at different distances (Figure 9). In this project, we also tested the influence of a different number of segments of the worm-like robot. The simulation shows that the difference between the paths of each segment increases with the number of segments (Figure 12). We also committed comparison experiments with physical robots. In the “straightforward” experiment, the physical robot′s real path was about 80% of the simulation result (Figure 14). In the offset clearance experiment, the physical robot’s path was 85.2% of the simulation result in the x-axis direction and 81.1% in the y-axis direction.

## Figures and Tables

**Table 1:** The ratios of the errors normalized by the diameter of the robots with 4, 6, and 8 segments. The ratios increasing with the increasing number of segments.

Path Error	4 Segments	6 Segments	8 Segments
L-Error	15.40%	17.80	20.20%
R-Error	9.60%	14.20%	17.90%

## Data Availability

Not applicable.

## Appendix A

**Steps for the path generation in an unknown situation**

Input: Acquire the initial and desired configuration of the robot, the position of the obstacles, and the maximum number of samples, *Nmax*.
Output: Bézier curve Path. 1. Set the center of the robot’s head as the starting point and set the ending point as the ending point. 2. For *i* = 1 to *Nmax*. 2.1 Set Bézier points of the first part and generate the path. - $P_{1}$—Current point of the robot’s head. - $P_{2}$—Current tangent direction of the robot’s head with random length $L_{1}$. - $P_{3}$—Current tangent direction of the desired direction with random length $L_{2}$. - $P_{4}$—Random point in the whole space to search the entrance of the passage. 2.2 Set Bézier points of the second part and generate the path. - $P_{1}$—First part’s $P_{4}$. - $P_{2}$—Current tangent direction of the first part’s $P_{3}$ with random length $L_{3}$. - $P_{3}$—Current tangent direction of the goal point with random length $L_{4}$. - $P_{4}$—Goal point. 2.3 Conduct collision detection for the Bézier curve path. 2.4 The robot’s head following the path and generate the path of each segment. 2.5 Conduct collision detection for the path of each segment. 2.6 Return no collision paths. 3. Choose the final path. - If the shortest path is chosen: Calculate the arc length of the paths. Return the shortest path. - If the path with the minimum number of waves is chosen: Calculate the numbers of waves of each path. Return the path with the minimum number of waves. 4. Return the final path.

**Steps for the path generation in a known situation**

Input: Get the initial and desired configuration of the robot and the position of the obstacles.
Output: Bézier curve Path. 1. Set the center of the robot’s head as the starting point and the ending point as the ending point. 2. Set Bézier points of the first part and generate the path. - $P_{1}$—Current point of the robot’s head. - $P_{2}$—Current tangent direction of the robot’s head with random length $L_{1}$. - $P_{3}$—Current tangent direction of the desired direction with random length $L_{2}$. - $P_{4}$—Specified point, its position, and direction are based on the position of the obstacles and passage. 3. Set Bézier points of the second part and generate the path. - $P_{1}$—First part’s $P_{4}$. - $P_{2}$—Current tangent direction of the first part’s $P_{3}$ with random length $L_{3}$. - $P_{3}$—Current tangent direction of the goal point with random length $L_{4}$. - $P_{4}$—Goal point. 4. Conduct collision detection for the Bézier curve path. 5. The robot’s head follows the path and generates the path of each segment. 6. Conduct collision detection for the path of each segment. 7. Choose the final path. - If the shortest path is chosen: Calculate the arc length of the paths. Return the shortest path. - If the path with the minimum number of waves is chosen: Calculate the numbers of waves of each path. Return the path with the minimum number of waves. 8. Return no collision paths.

## Appendix B

**Steps for the algorithm to find the turn clearance limitations**

Input: Initial and desired configuration of the robot; the distance of the obstacles; the initial offset of the obstacle, $Offset_{init}$; the maximum number of iterations, *Nmax*.
Output: Bézier curve Path and the offset of the largest obstacle. 1. Set the center of the robot’s head as the starting point, the center of the passage’s entrance as the ending point, and the center of the passage’s exit as the goal point. 2. While finding a Bézier curve that satisfies the constraints, let the robot successfully traverse the passage, Offset = Offset+1 cm. 2.1 For *i* = 1 to *Nmax*. 2.1.1 Set Bézier points of the first part and generate the path. - $P_{1}$—Current point of the robot’s head. - $P_{2}$—Current tangent direction of the robot’s head with random length $L_{1}$. - $P_{3}$—Current tangent direction of the desired direction with random length $L_{1}$. - $P_{4}$—The center of the passage’s entrance. 2.1.2 Set Bézier points of the second part and generate the path. - $P_{1}$—The center of the passage’s entrance. - $P_{2}$—Current tangent direction of the first part’s $P_{3}$ with random length $L_{2}$. - $P_{3}$—Current tangent direction of the goal point with random length $L_{2}$. - $P_{4}$—The center of the exit of the passage’s exit. 2.1.3 Conduct collision detection for the Bézier curve path. 2.1.4 Robot follows the path and generates the path of each segment. 2.1.5 Conduct collision detection for the path of each segment. 2.1.6 Return no collision paths. 2.2 Choose the final path. Calculate the arc length of the paths. Return the shortest path and continue the while looping. 3. Return the final path and the offset of the largest obstacle.

## References

[1] Zhang B., Fan Y., Yang P., Cao T., Liao H. (2019). Worm-Like Soft Robot for Complicated Tubular Environments. Soft Robot..

[2] Reina G., Ojeda L., Milella A., Borenstein J. (2006). Wheel slippage and sinkage detection for planetary rovers. IEEE/ASME Trans. Mechatronics.

[3] Kandhari A., Wang Y., Chiel H., Daltorio K.A. (2019). Turning in Worm-Like Robots: The Geometry of Slip Elimination Suggests Nonperiodic Waves. Soft Robot..

[4] Raja P., Pugazhenthi S. (2012). Optimal path planning of mobile robots: A review. Int. J. Phys. Sci..

[5] LaValle S.M. (2006). Planning Algorithms.

[6] Wang Y., Pandit P., Kandhari A., Liu Z., Daltorio K.A. (2020). Rapidly Exploring Random Tree Algorithm-Based Path Planning for Worm-Like Robot. Biomimetics.

[7] Hu B., Cao Z., Zhou M. (2021). An Efficient RRT-Based Framework for Planning Short and Smooth Wheeled Robot Motion Under Kinodynamic Constraints. IEEE Trans. Ind. Electron..

[8] Kang J.-G., Lim D.-W., Choi Y.-S., Jang W.-J., Jung J.-W. (2021). Improved RRT-Connect Algorithm Based on Triangular Inequality for Robot Path Planning. Sensors.

[9] Hassani V., Lande S.V. (2018). Path Planning for Marine Vehicles using Bézier Curves. IFAC-PapersOnLine.

[10] Choi J.-W., Curry R., Elkaim G. Path Planning Based on Bézier Curve for Autonomous Ground Vehicles. Proceedings of the Advances in Electrical and Electronics Engineering—IAENG Special Edition of the World Congress on Engineering and Computer Science 2008.

[11] Jollyb K.G., Sreerama Kumar R., Vijayakumara R. (2009). A Bézier curve based path planning in a multi-agent robot soccer system without violating the acceleration limits. Robot. Auton. Syst..

[12] Baydas S., Karakas B. (2019). Defining a curve as a Bezier curve. J. Taibah Univ. Sci..

[13] JGreer J.D., Blumenschein L.H., Okamura A.M., Hawkes E.W. Obstacle-Aided Navigation of a Soft Growing Robot. Proceedings of the 2018 IEEE International Conference on Robotics and Automation (ICRA).

[14] Dutta A.K., Debnath S.K., Das S.K. (2019). Path-Planning of Snake-Like Robot in Presence of Static Obstacles Using Critical-SnakeBug Algorithm. Advances in Computer, Communication and Control.

[15] Zarrouk D., Sharf I., Shoham M. Analysis of earthworm-like robotic locomotion on compliant surfaces. Proceedings of the 2010 IEEE International Conference on Robotics and Automation.

[16] Kandhari A., Daltorio K.A. A kinematic model to constrain slip in soft body peristaltic locomotion. Proceedings of the 2018 IEEE International Conference on Soft Robotics (RoboSoft).

[17] Kandhari A. (2020). Control and Analysis of Soft Body Locomotion on a Robotic Platform. Electronic Ph.D. Dissertation.

[18] Horchler A.D., Kandhari A., Daltorio K.A., Moses K.C., Ryan J.C., Stultz K.A., Kanu E.N., Andersen K.B., Kershaw J.A., Bachmann R.J. (2015). Peristaltic Locomotion of a Modular Mesh-Based Worm Robot: Precision, Compliance, and Friction. Soft Robot..

[19] Chowdhury A., Ansari S., Bhaumik S. (2017). Earthworm like modular robot using active surface gripping mechanism for peristaltic locomotion. Proceedings of the Advances in Robotics (AIR ‘17).

[20] Gough E., Conn A.T., Rossiter J. (2021). Planning for a Tight Squeeze: Navigation of Morphing Soft Robots in Congested Environments. IEEE Robot. Autom. Lett..

[21] Kandhari A., Wang Y., Chiel H.J., Quinn R.D., Daltorio K.A. (2021). An Analysis of Peristaltic Locomotion for Maximizing Velocity or Minimizing Cost of Transport of Earthworm-Like Robots. Soft Robot..

[22] Trivedi D., Rahn C.D., Kier W.M., Walker I.D. (2008). Soft Robotics: Biological Inspiration, State of the Art, and Future Research. Appl. Bionics Biomech..

[23] Polygerinos P., Correll N., Morin S.A., Mosadegh B., Onal C.D., Petersen K., Cianchetti M., Tolley M.T., Shepherd R.F. (2017). Soft Robotics: Review of Fluid-Driven Intrinsically Soft Devices; Manufacturing, Sensing, Control, and Applications in Human-Robot Interaction. Adv. Eng. Mater..

[24] Ariff M., Zamzuri H., Nordin M., Yahya W., Mazlan S.A., Rahman M. (2015). Optimal Control Strategy for Low Speed and High Speed Four-Wheel-Active Steering Vehicle. J. Mech. Eng. Sci..

[25] Kandhari A., Huang Y., Daltorio K., Chiel H., Quinn R. (2018). Body stiffness in orthogonal directions oppositely affects worm-like robot turning and straight-line locomotion. Bioinspir. Biomim..

[26] Horchler A.D., Kandhari A., Daltorio K.A., Moses K.C., Andersen K.B., Bunnelle H., Kershaw J., Tavel W.H., Bachmann R.J., Chiel H.J., Wilson S.P., Verschure P.F., Mura A., Prescott T.J. (2015). Worm-like robotic locomotion with a compliant modular mesh. Living Machines 2015.

[27] Hernandez B., Giraldo E. A Review of Path Planning and Control for Autonomous Robots. Proceedings of the 2018 IEEE 2nd Colombian Conference on Robotics and Automation (CCRA).

[28] Coad M.M., Blumenschein L.H., Cutler S., Zepeda J.A.R., Naclerio N.D., El-Hussieny H., Mehmood U., Ryu J.-H., Hawkes E.W., Okamura A.M. (2019). Vine Robots: Design, Teleoperation, and Deployment for Navigation and Exploration. IEEE Robot. Autom. Mag..

[29] Kandhari A., Stover M.C., Jayachandran P.R., Rollins A., Chiel H.J., Quinn R.D., Daltorio K.A. (2018). Distributed Sensing for Soft Worm Robot Reduces Slip for Locomotion in Confined Environments. Biomim. Biohybrid Syst..

[30] An D., Wang H. VPH: A new laser radar based obstacle avoidance method for intelligent mobile robots. Proceedings of the Fifth World Congress on Intelligent Control and Automation.

[31] Mattamala M., Ramezani M., Camurri M., Fallon M. Learning Camera Performance Models for Active Multi-Camera Visual Teach and Repeat. Proceedings of the IEEE International Conference on Robotics and Automation (ICRA).

[32] Transeth A.A., Leine R.I., Glocker C., Pettersen K.Y., Liljebäck P. (2008). Snake Robot Obstacle-Aided Locomotion: Modeling, Simulations, and Experiments. IEEE Trans. Robot..

